# Contrast-enhanced ultrasound characteristics of preoperative central cervical lymph node metastasis in papillary thyroid carcinoma

**DOI:** 10.3389/fendo.2022.941905

**Published:** 2022-09-05

**Authors:** Fei Ye, Yi Gong, Kui Tang, Yan Xu, Rongsen Zhang, Sijie Chen, Xiaodu Li, Qi Zhang, Liyan Liao, Zhongkun Zuo, Chengcheng Niu

**Affiliations:** ^1^ Department of General Surgery, The Second Xiangya Hospital, Central South University, Changsha, China; ^2^ Department of Ultrasound Diagnosis, The Second Xiangya Hospital, Central South University, Changsha, China; ^3^ Research Center of Ultrasonography, The Second Xiangya Hospital, Central South University, Changsha, China; ^4^ Department of Pathology, The Second Xiangya Hospital, Central South University, Changsha, China

**Keywords:** papillary thyroid carcinomas, contrast-enhanced ultrasound, conventional ultrasound, lymph node ultrasonography, central cervical lymph node metastasis

## Abstract

This study evaluated the preoperative diagnostic value of lymph node ultrasonography in distinguishing between benign and malignant central cervical lymph nodes (CCLNs) in patients with papillary thyroid carcinoma (PTC). A total of 176 patients who had PTC with 216 CCLNs (49 benign and 155 malignant) were enrolled in this study and preoperatively imaged by ultrasonography, including conventional ultrasound (US) and contrast-enhanced ultrasound (CEUS). We evaluated the ultrasonography parameters for each lymph node. Binary logistic regression analysis indicated that multifocality of PTC and the absence of Hashimoto’s thyroiditis are independent clinical features related to patients with PTC who also have malignant CCLNs. For preoperative ultrasonography features, heterogeneous enhancement and centripetal perfusion are independent ultrasonographic features to identify malignant and benign CCLNs. This study demonstrated that preoperative CEUS characteristics help to distinguish malignant CCLNs from benign CCLNs.

## Introduction

Papillary thyroid carcinoma (PTC) is the most common type of thyroid cancer with a high incidence of central or lateral cervical lymph node metastasis (1). The presence of cervical lymph node metastasis is a risk factor for locoregional recurrence and distant metastases (2–5). Conventional ultrasound (US) is the recommended imaging modality for assessing lymph nodes in patients with proven thyroid cancers (6, 7). However, the US sensitivity for detecting malignant lymph nodes is relatively low, especially in the central compartment, due to the presence of the overlying thyroid gland and nodal micrometastases (size ≤2 mm in diameter) (6, 8). However, there is no significant association between nodal micrometastases and the risk of recurrence; thus, most of the nodal micro-metastases that go undetected by US are of little clinical significance (9, 10). Therefore, preoperatively identified macroscopic metastatic lymph nodes (size >2 mm in diameter) present important prognostic significance for a high risk of recurrence (11, 12).

The central cervical lymph nodes (CCLNs) are commonly referred to as level VI lymph nodes, which include the prelaryngeal, pretracheal, left paratracheal, and right paratracheal lymph nodes (13, 14). However, due to the low sensitivity of CCLNs, most of the studies on CCLN metastasis were based on the predictive values of conventional US features of PTC nodules, and few studies reported the ultrasonographic characteristics of the CCLNs themselves (15–18).

Contrast-enhanced ultrasound (CEUS) has high sensitivity for vascularity compared with color Doppler imaging. CEUS has been applied to identify malignant and benign lymph nodes with good performance (19, 20). However, most of the studies associated with the US features of metastatic lymph nodes are based on those of lateral cervical lymph nodes, and some of those reviews were not applicable to CCLNs (19–22). Thus, we are eager to determine the US features of CCLNs, especially macroscopic metastatic lymph nodes, which have considerable significance in designing individualized surgical approaches. However, to the best of our knowledge, the US features of CCLNs, especially the capability of CEUS for identifying metastatic CCLNs from PTC, have rarely been reported. Here, we evaluated the ultrasonographic features and preoperative value of metastatic CCLNs from PTC.

## Methods and materials

### Patients

The study was approved by the Ethical Committee of the Second Xiangya Hospital of Central South University in China. From May 2021 to November 2021, 521 consecutive patients with PTC with or without CCLN metastasis (296 patients with CCLN metastasis and 225 patients without CCLN metastasis) were retrospectively enrolled in this study. (i) CCLNs that received conventional US and CEUS examinations and (ii) a long-axis diameter of CCLNs ≥3 mm were included. CCLNs without surgical pathologic results were excluded from this study. Finally, 176 patients with PTC and 216 CCLNs (61 benign and 155 malignant) were finally included. Additionally, thyroid-related functional laboratory results, including free T4, free T3, thyroid peroxidase antibody (A-TPO), and thyroglobulin antibody (A-TG), were obtained within one week of surgery in all patients. The clinical parameters of the patients, including age, sex, Hashimoto’s thyroiditis, BRAF ^V600E^ mutation, multifocality, and TNM stage, were recorded (23).

### Conventional US and CEUS examinations

All selected CCLNs were imaged by conventional US (Siemens S3000, Mountain View, CA, USA) for the following US features: the largest diameter, long axis/short axis ratio (L/S) ≥2 or <2, presence or absence of fatty hilum, hyperechogenicity, microcalcification, and cystic change. CEUS was performed with a SonoVue (Bracco, Italy) intravenous bolus injection of 2.4 ml. The video of the CEUS was recorded for more than 30 s to image the lymph node *via* the vein lasting approximately 30 s. Compared with surrounding adjacent tissue enhancement, the contrast enhancement qualitative characteristics were classified as follows: hyper-, iso-, hypo-, or non-enhancement type (hyper-enhancement means as high as the thyroid tissue; hypo-enhancement means equal to or lower than the surrounding adipose tissue; iso-enhancement was between them), heterogeneous or homogeneous enhancement homogeneity, centripetal or centrifugal perfusion pattern, presence or absence of ring enhancement. Compared with surrounding adjacent adipose tissue enhancement, the contrast enhancement quantitative characteristics were classified as follows: peak intensity (PI) index >1 or ≤1, and area under the curve (AUC) index >1 or ≤1 (20).

### Reference standard

A diagnosis of malignant or benign CCLNs was confirmed by the histopathological results after surgery.

### Statistical analysis

Statistical analysis was performed using SPSS version 21.0 software (SPSS, Chicago, IL, USA). Independent t and chi-square tests were used for continuous data and categorical data, respectively. Binary logistic regression was used to evaluate significant clinical or ultrasonographic characteristics and their independent association with CCLN metastasis. *p <*0.05 was determined to be statistically significant.

## Results

**Figure 1:**
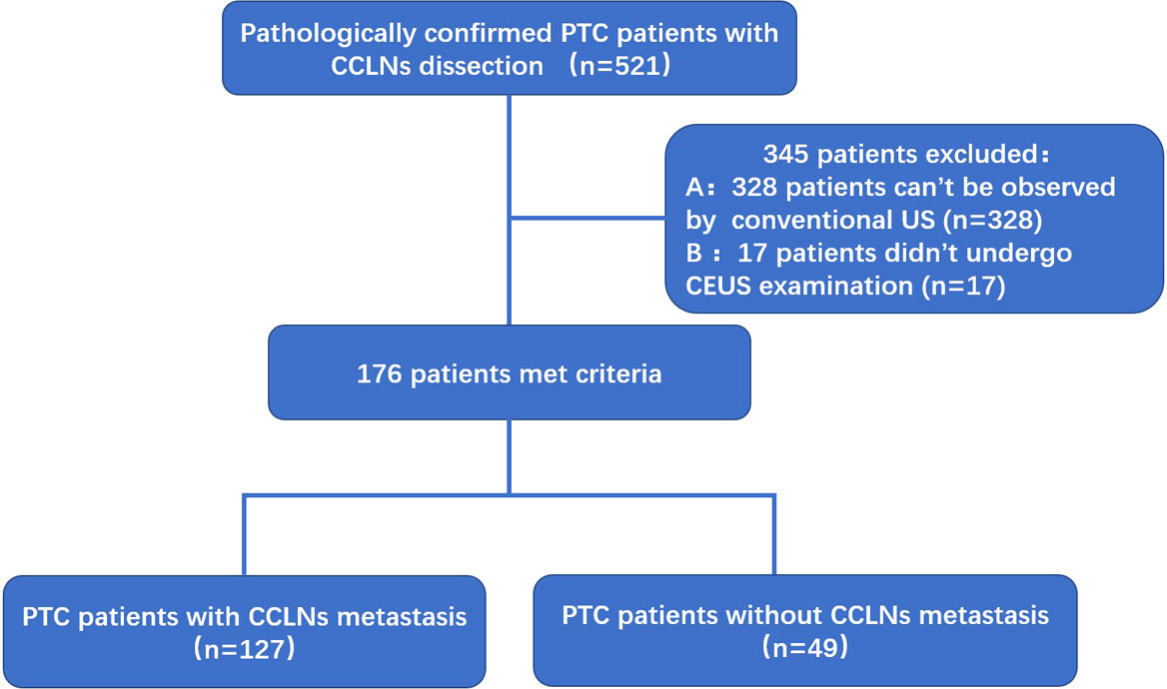
Flowchart for the selection of patients with PTC. PTC, papillary thyroid carcinoma; CCLNs, Central cervical lymph nodes.

In this study, 521 consecutive patients with PTC with or without CCLN metastases (296 patients with CCLN metastasis and 225 patients without CCLN metastasis) were retrospectively enrolled. Of the 296 patients with PTC with CCLN metastasis, 144 patients with CCLNs were observed by preoperative US, and 152 patients with CCLNs could not be observed by preoperative US. Thus, the diagnosis of the presence of CCLNs was 48.6% in the 296 patients with PTC with CCLN metastasis by preoperative US. Otherwise, of the 225 patients with PTC without CCLN metastasis, only 49 patients with CCLNs could be observed by preoperative US, and 176 patients without CCLN metastasis could not be observed by preoperative US. Thus, the diagnosis of the presence of CCLNs was only 21.8% in the 225 patients with PTC without CCLN metastasis by preoperative US. Therefore, the total diagnosis of the presence of CCLNs was 37.0% in 521 patients by preoperative US in this study, and 328 patients were excluded because there was no detection of CCLNs by conventional US. However, the diagnosis of the presence of CCLNs in the malignant CCLN group was as high as 74.6% for the 193 patients with CCLNs observed by preoperative US. Of the 193 patients with CCLNs observed, 17 patients with CCLNs who only underwent conventional US were not enrolled. Finally, 176 patients with PTC (49 benign and 127 malignant) and 216 CCLNs (61 benign and 155 malignant) were included (**Figure 1**).

**Table 1** shows the clinical features of the patients with PTC with malignant and benign CCLNs. Among the 176 patients with PTC, 127 patients had CCLN metastasis from PTC, and 49 patients only had benign CCLNs after CCLN dissection. The average age of patients with PTC with CCLN metastasis was 37.40 ± 11.36 years (range: 18–69 years) and that of patients with benign CCLN was 39.29 ± 9.54 years (range: 21–55 years); there was no difference between them (p = 0.305). A total of 130 (73.9%) patients with PTC were female, and 46 (26.1%) patients with PTC were male. However, 31.5% of patients with CCLN metastasis were male and only 12.2% of patients without CCLN metastasis were male (p = 0.009), showing that males were more likely to have CCLN metastasis. Sixty-seven (52.8%) patients with CCLN metastasis had Hashimoto’s thyroiditis, and 45 (91.8%) patients without CCLN metastasis had Hashimoto’s thyroiditis (p = 0.000), indicating that Hashimoto’s thyroiditis is a protective factor for hindering CCLN metastasis in patients with PTC. There were 105 (82.7%) patients with PTC with CCLN metastases and 41 (83.8%) patients with PTC without CCLN metastasis who had BRAF^V600E^ mutations (p = 0.781), and there was no difference between them. Sixty-one (48.0%) patients with CCLN metastasis and 10 (20.4%) patients without CCLNs had multifocal PTCs (p = 0.001), demonstrating that the multifocality of PTC was more inclined to have CCLN metastasis. Among the 127 patients with CCLN metastases from PTC, 120 patients were in TNM stage I, and seven patients were in TNM stage II. In the 49 patients with benign CCLNs from PTC, all the patients were in TNM stage I, and there was no difference between them (p = 0.193).

**Table 1:** Clinical features of the patients with malignant and benign CCLNs.

Features	Malignant CCLN patients (n = 127)		Benign CCLN patients (n = 49)	*P*-Value
Age (y)	37.40 ± 11.36		39.29 ± 9.54	0.305
Male sex	40 (31.5)		6 (12.2)	0.009*
Hashimoto’s thyroiditis	67 (52.8)		45 (91.8)	0.000*
BRAF^V600E^ mutation Multifocality TNM stage I/II	105 (82.7) 61 (48.0) 120/7		41 (83.8) 10 (20.4) 49/0	0.781 0.001* 0.193

*p <0.05 was considered a significant difference.

**Figure 2:**
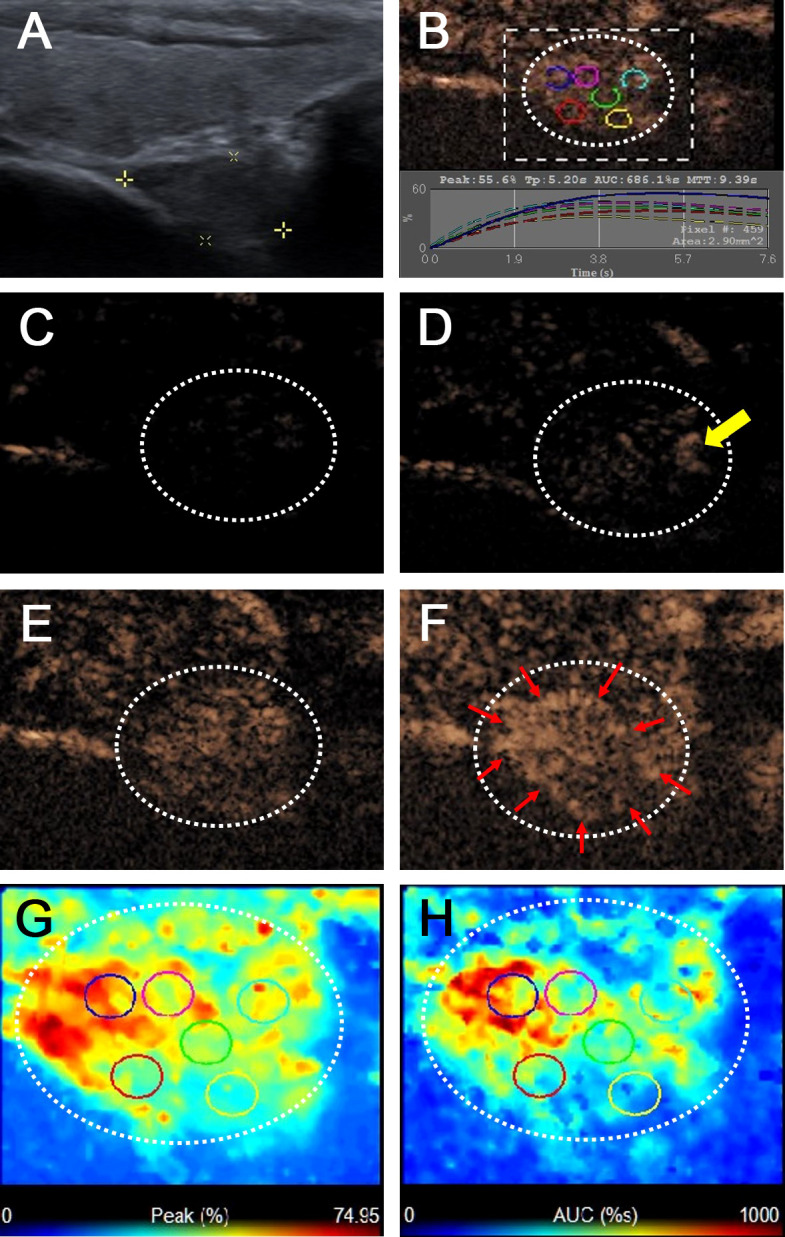
Metastatic lymph node in a 38-year-old female patient with PTC. **(A)** The metastatic foci in a right paratracheal CCLN (white cross) identified on conventional US, showing L/S <2, absence of fatty hilum and hyperechoic appearance. **(B)** Time-intensity curve of CCLN. **(C–F)** CEUS images at different times, **(C)** before injection of contrast agent, the CCLN showed no microbubbles signal; **(D)** at 9 s, the CCLN showed the microbubbles signal from the periphery of the lymph node (yellow arrow); **(E)** at 11 s, the whole CCLN showed heterogeneous enhancement, which is equal to the surrounding thyroid tissue; **(F)** at 13 s, the whole CCLN showed heterogeneous hyper-enhancement with a ring enhancement (red arrows), which is higher than the surrounding thyroid tissue. **(G, H)** Parametric color maps of PI and AUC values, PI and AUC values of the whole lymph nodes (yellow and red colors) was higher than the surrounding tissue (blue color), means PI index >1 and AUC index >1, **(G)** PI values for the periphery of CCLN were higher than those of the center of CCLN, which were heterogeneous of the whole CCLN; **(H)** AUC values for the periphery of CCLN were higher than those of the center of CCLN, which were also heterogeneous of the whole CCLN.

**Figure 3:**
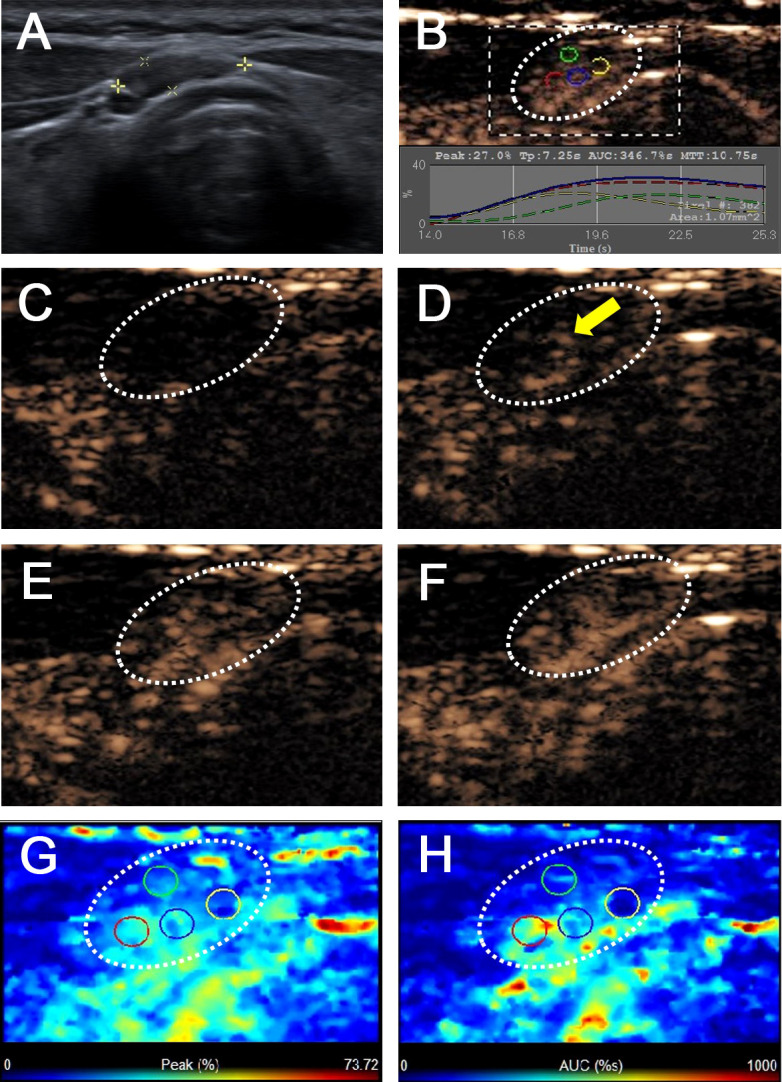
Benign lymph node in a 43-year-old female patient with PTC with Hashimoto’s thyroiditis. **(A)** A pretracheal CCLN with the absence of fatty hilum (white cross) identified on conventional US. **(B)** Time-intensity curve of CCLN. **(C–F)** CEUS images at different times, **(C)** before injection of contrast agent, the CCLN showed no microbubbles signal; **(D)** at 10 s, the CCLN showed the microbubbles signal from the center of the lymph node (yellow arrow); **(E)** at 14 s, the whole CCLN showed homogeneous enhancement, which is equal to the surrounding thyroid tissue; **(F)** at 16 s, the whole CCLN showed homogeneous iso-enhancement, which is equal to the surrounding thyroid tissue. **(G, H)** Parametric color maps of PI and AUC values, PI and AUC values of the whole lymph nodes (cyan and blue colors) was equal to the surrounding tissue (cyan and blue colors), means PI index ≤1 and AUC index ≤1, **(G)** PI values for the center of CCLN were higher than those of the periphery of CCLN, which were homogeneous of the whole CCLN; **(H)** AUC values for the center of CCLN were higher than those of the periphery of CCLN, which were also homogeneous of the whole CCLN.

**Figure 4:**
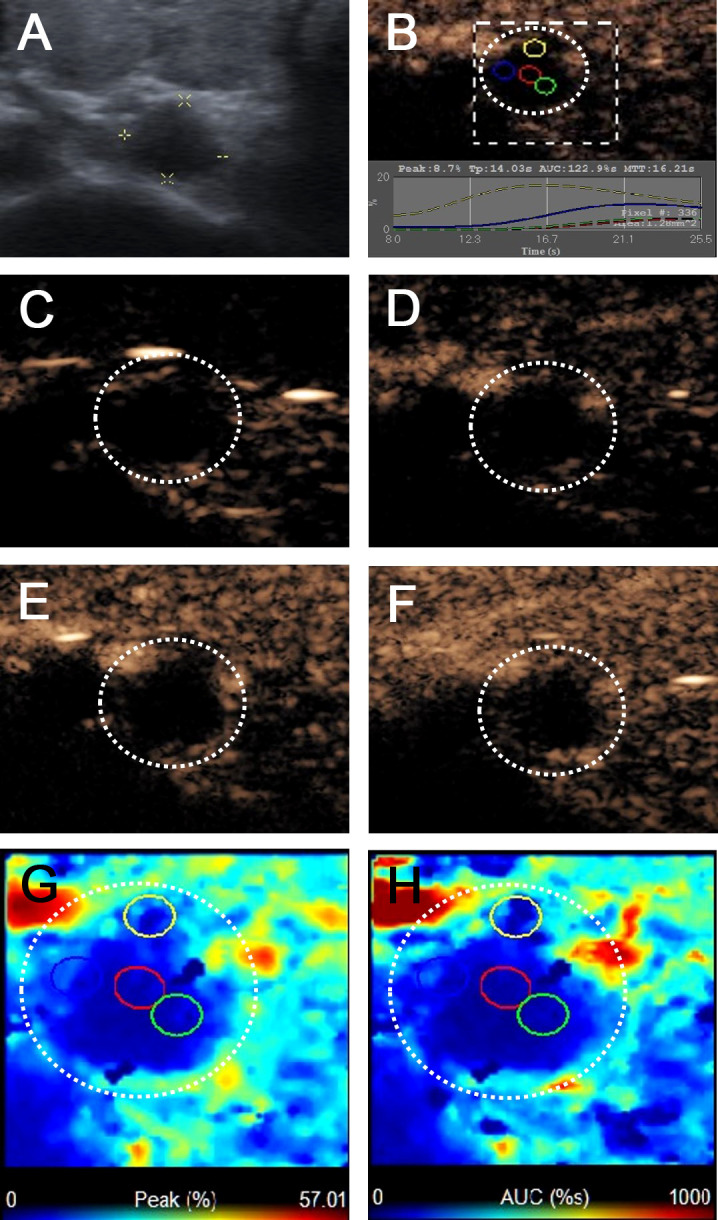
Metastatic lymph node in a 69-year-old female patient with PTC and Hashimoto’s thyroiditis. **(A)** The metastatic foci in a right paratracheal CCLN (white cross) identified on conventional US, showing L/S <2 and absence of fatty hilum appearance. **(B)** Time-intensity curve of CCLN. **(C–F)** CEUS images showed no microbubbles signal in the CCLN at different times **(C)** before injection of contrast agent, **(D)** 10 s, **(E)** 12 s, **(F)** 15 s after injection of contrast agent. **(G, H)** Parametric color maps of PI and AUC values, PI and AUC values of the whole lymph nodes (blue color) was lower than the surrounding tissue (cyan and red colors), means PI index ≤1 and AUC index ≤1.

**Table 2** summarizes the ultrasonographic features of the malignant and benign CCLNs in patients with PTC. The mean diameter of malignant CCLNs was 9.18 ± 3.92 mm (range: 3–25 mm), and that of benign CCLNs was 8.38 ± 2.91 mm (range: 5–19 mm); there was no difference between them (p = 0.102). Four conventional US signs (including L/S <2, a fatty hilum, hyperechogenic appearance, and calcification) were of diagnostic value for the identification of malignant CCLNs from PTCs (all p <0.05, **Figures 2**–**4**). However, a cystic change was found only in 7.7% of the malignant CCLNs and 1.6% of the benign CCLNs (p = 0.090), which was not a statistically significant difference in this study. For CEUS parameters, six CEUS features (including hyper-enhancement, heterogeneous enhancement, centripetal perfusion, ring enhancement, PI index >1, and AUC index >1) were of diagnostic value for malignant CCLNs from PTCs (all p <0.05, **Figures 2**, **4**).

**Table 2:** Ultrasonographic features of the malignant and benign CCLNs in patients with PTC.

Features	Malignant CCLNs (n = 155)	Benign CCLNs (n = 61)	*P*-Value
**Conventional US parameters** Size (mm)	9.18 ± 3.92	8.38 ± 2.91	0.102
L/S <2 ≥2 Fatty hilum Present Absent Hyperechogenicity Present Absent Calcification Present Absent Cystic change Present Absent **CEUS parameters** Enhancement type Hyper- Hypo- or iso- Non- Enhancement homogeneity Homogeneous Heterogeneous Perfusion Centripetal Centrifugal Ring enhancement Present Absent PI index >1 ≤1	119 (76.8) 36 (23.2) 4 (2.6) 151 (97.4) 71 (45.8) 84 (54.2) 59 (38.1) 96 (61.9) 12 (7.7) 143 (92.3) 113 (72.9) 38 (24.5) 4 (2.6) 65 (41.9) 90 (58.1) 127 (81.9) 28 (18.1) 65 (41.9) 90 (58.1) 113 (72.9) 42 (27.1)	31 (50.8) 30 (49.2) 8 (13.1) 53 (86.9) 4 (6.6) 57 (93.4) 7 (11.5) 54 (88.5) 1 (1.6) 60 (98.4) 8 (13.1) 52 (85.3) 1 (1.6) 60 (98.4) 1 (1.6) 3 (4.9) 58 (95.1) 4 (6.6) 57 (93.4) 8 (13.1) 53 (86.9)	0.000* 0.002* 0.000* 0.000* 0.090 0.000* 0.000* 0.000* 0.000* 0.000*
AUC index >1 ≤1	113 (72.9) 42 (27.1)	8 (13.1) 53 (86.9)	0.000*

L/S, ratio of long axis to short axis; PI, peak intensity; AUC, area under the curve.

*p <0.05 was considered a significant difference.

Binary logistic regression analysis was performed for all of the statistically significant clinical or ultrasonographic characteristic variables. The clinical results indicated that Hashimoto’s thyroiditis (β = −2.393, odds ratio [OR] = 0.091, 95% confidence interval [CI] = 0.030–0.279, p = 0.000) was an independent clinical feature related to patients with PTC with benign CCLNs, and thyroid cancer multifocality (β = 1.544, odds ratio [OR] = 4.684, 95% confidence interval [CI] = 2.019–10.869, p = 0.000) was an independent clinical feature related to patients with PTC with malignant CCLNs (**Table 3**). The ultrasonographic results indicated that heterogeneous enhancement (β = 2.775, odds ratio [OR] = 16.032, 95% confidence interval [CI] = 1.919–133.954, p = 0.010) and centripetal perfusion enhancement (β = 3.630, odds ratio [OR] = 37.722, 95% confidence interval [CI] = 10.606–134.160, p = 0.000) were independent US features related to malignant CCLNs distinguishing them from benign CCLNs (**Table 4**).

**Table 3:** Multivariate logistic regression analysis of clinical features related to the patients with PTC with malignant CCLNs.

Features	Partial regression coefficient, β	Odds ratio	95% Confidence interval	*P*-Value
Hashimoto’s thyroiditis	−2.393	0.091	0.030–0.279	0.000*
Multifocality	1.544	4.684	2.019–10.869	0.000*

*p <0.05 was considered a significant difference.

**Table 4:** Multivariate logistic regression analysis of ultrasonographic features related to malignant CCLNs distinguishing from benign CCLNs.

Features	Partial regressioncoefficient, β	Odds ratio	95% Confidence interval	*P*-Value
Heterogeneous enhancement	2.775	16.032	1.919–133.954	0.010*
Centripetal perfusion	3.630	37.722	10.606–134.160	0.000*

*p <0.05 was considered a significant difference.

As heterogeneous enhancement and centripetal perfusion enhancement were the independent characteristics related to malignant CCLNs for differentiating them from benign CCLNs, we chose these CEUS parameters to calculate the diagnostic accuracy of malignant CCLNs using two methods. Method one (CEUS 1): if the CCLN had one of these CEUS parameters, the CCLN was classified as malignant, while if the CCLN had none of these CEUS parameters, the CCLN was classified as benign. Method two (CEUS 2): if the CCLN had two of these CEUS parameters, the CCLN was classified as malignant, while if the CCLN had none or only one of these CEUS parameters, the CCLN was classified as benign. Thus, the diagnostic performance of the two different methods for differentiating between benign and malignant CCLNs is outlined in **Table 5**. The CEUS1 method achieved an Az value of 0.890 with an accuracy of 87.0% (188/216), whereas the CEUS2 method achieved an Az value of 0.777 with an accuracy of 68.1% (147/216). Whether compared with the Az values or accuracy, the CEUS1 method had better diagnostic value than the CEUS2 method.

**Table 5:** Diagnostic performance for discrimination between benign and malignant CCLNs.

Methods	Sensitivity (%)	Specificity (%)	Accuracy (%)	PPV (%)	NPV (%)	Az (95% CI)
CEUS1	84.5	93.4	87.0	97.0	70.4	0.890 (0.840–0.939)
CEUS2	55.5	100	68.1	100	46.9	0.777 (0.718–0.937)

CEUS, contrast-enhanced ultrasound; CI, confidence interval; PPV, positive predictive value; NPV, negative predictive value.

## Discussion

High-resolution ultrasonography is the recommended imaging modality for the preoperative diagnosis of PTC and cervical lymph nodes (6). Recent studies have shown that the US features of PTC may also be useful in predicting cervical lymph node metastasis in patients with PTC (24–26). In this study, we directly reviewed the US characteristics of macroscopic CCLNs (size ≥3 mm in diameter) and evaluated the preoperative value of conventional US and CEUS in metastatic CCLNs from PTC.

In this study, male sex was found to be a clinical risk factor for CCLN metastasis in patients with PTC, which is consistent with previous findings (27–29). However, other studies have found that there is no association between sex and CCLN metastasis (24, 25). Thus, the association between sex and CCLN metastasis in PTC remains controversial. In this study, Hashimoto’s thyroiditis was found to be an independent clinical characteristic related to patients with PTC with benign CCLNs, which was also reported in previous studies showing that the presence of Hashimoto’s thyroiditis is a protective factor for a lower incidence of cervical lymph node metastasis in patients with PTC (30–32). Another finding was that thyroid cancer multifocality is an independent clinical characteristic related to patients with PTC with metastatic CCLNs, which is consistent with some previous reports (16, 33). In our study, the multifocality rate of PTCs was found to be 48.0% in the malignant CCLN group, which was also close to that reported in the literature (16, 33).

According to the clinical guidelines, metastatic cervical lymph nodes often show a round shape, the absence of a fatty hilum, hyperechogenicity, microcalcifications, and cystic changes (6, 34). In this study, metastatic CCLNs with a round shape (L/S <2) were found in 76.8% of malignant CCLNs and 50.8% of benign CCLNs, providing a sensitivity of 76.8% and a specificity of 49.2%, whose sensitivity was much higher than that of 37.0%–44.6% and the sensitivity was much lower than that of 70.0%–72.7% in the reported literature (6, 20, 34). This result indicates that lymph nodes of a round shape are more frequently imaged in the central cervical compartment than in the lateral cervical compartment. Otherwise, more than half of the patients included in this study had Hashimoto’s thyroiditis, especially in the benign CCLN group, which is usually accompanied by swollen lymph nodes in the central compartment. A fatty hilum was absent in 97.4% of the malignant CCLNs and in 86.9% of the benign CCLNs, providing a sensitivity of 97.4% and a specificity of 13.1%, whose sensitivity was close to that of 91.1%–99.5% (19, 20, 34), and the sensitivity was much lower than that of 22.7%–51.0% reported in the literature (19, 20). This result may contribute to the selection bias of many patients with Hashimoto’s thyroiditis included in this study. A hyperechogenic appearance was found in 45.8% of the malignant CCLNs but only 6.6% of the benign CCLNs, providing a sensitivity of 45.8% and a specificity of 93.4%; its sensitivity and specificity were in the range of 30.0%–87.0% and 43.0%–95.0%, respectively, in the reported literature (6, 34). Calcification was present in 38.1% of the malignant CCLNs but only 11.5% of the benign CCLNs, providing a sensitivity of 38.1% and a specificity of 88.5%. Its sensitivity was in the range of 5.0%–69.0% in the reported literature, while its sensitivity was lower than that of 93.0%–100.0% in the reported literature (6, 19, 34). In this study, cystic changes were only present in 7.7% of the malignant CCLNs and 1.6% of the benign CCLNs, providing a sensitivity of 7.7% and a specificity of 98.4%; in the reported literature, its sensitivity was lower than that of 10.0%–34.0%, and its specificity was in the range of 91.0%–100.0% (6, 19, 20, 34).

Recently, some studies have shown that CEUS features are helpful for the preoperative diagnosis of cervical lymph nodes in patients with PTC. Chen et al. found that cervical lymph node metastasis from PTC showed centripetal perfusion, peripheral or mixed enhancement, and an enlarged range on CEUS compared with the initial size of the US (21). Our previous study demonstrated that hyper-enhancement, centripetal perfusion, and ring enhancement are independent CEUS characteristics that can be used to identify malignant and benign cervical lymph nodes in patients with PTC (20). However, most of these findings are based on the CEUS features of lateral cervical lymph nodes, not from the CCLNs. Thus, it is important to investigate the CEUS features of macroscopic CCLNs to design individualized surgical treatment strategies.

In this study, malignant CCLNs from PTCs more often showed hyper-enhancement, heterogeneous enhancement, centripetal perfusion, ring enhancement, PI index >1 and AUC index >1 than benign CCLNs. A hyper-enhancement was found in 72.9% of the malignant CCLNs but only 13.1% of the benign CCLNs, providing a sensitivity of 72.9% and a specificity of 86.9%. Both a PI index >1 and an AUC index >1 were shown in 72.9% of the malignant CCLNs and 13.1% of the benign CCLNs, providing a sensitivity of 72.9% and a specificity of 86.9%. These results were consistent with our previous study on metastatic cervical lymph nodes, which consisted of most lateral lymph nodes and parts of CCLNs, indicating that hyper-enhancement was a common CEUS characteristic of both lateral and central cervical lymph nodes due to focal neovascularization from tumor cells (20). Heterogeneous enhancement was shown in 58.1% of the malignant CCLNs but only 1.6% of the benign CCLNs, providing a sensitivity of 58.1% and a specificity of 98.4%, whose sensitivity was higher than that of 53.1% in the study by Hong et al. and lower than that of 72.0% in our previous study. However, its specificity was much higher than that of the other two studies (19, 20). This result demonstrated that heterogeneous enhancement was observed in more than half of the metastatic CCLNs on CEUS due to the immature neovascularization and avascular necrotic areas in metastatic foci, while almost all the benign CCLNs manifested homogeneous enhancement. For the CEUS perfusion pattern, centripetal perfusion was found in 81.9% of the malignant CCLNs but only in 4.9% of the benign CCLNs, providing a sensitivity of 81.9% and a specificity of 95.1%, which means that the input lymphatic vessels and subcapsular sinuses were invaded by tumor cells in most of the metastatic CCLNs; thus, the microbubbles spread from the periphery to the inner lymph area, presenting as centripetal enhancement on CEUS. These results are consistent with previous studies (20, 21). Furthermore, ring enhancement was present in 41.9% of the malignant CCLNs but only 6.6% of the benign CCLNs in this study, providing a sensitivity of 41.9% and a specificity of 93.4%, whose sensitivity was much lower than that of 60.1% in our previous study, indicating that ring enhancement was observed less frequently than that in lateral cervical lymph nodes (20). Ring enhancement is likely caused by the capsular and peripheral vessels around the lymph node, which is the last area to be invaded by tumor cells in the metastatic foci (19, 20).

According to the results of binary logistic regression analysis, heterogeneous enhancement and centripetal perfusion enhancement were the independent characteristics related to malignant CCLNs, which can be used to differentiate them from benign CCLNs. These CEUS parameters were used to calculate the diagnostic accuracy of malignant CCLNs with an Az value of 0.890 and an accuracy of 87.0% by the CEUS1 method, which provides a potential diagnostic basis for the diagnosis of benign and malignant CCLNs.

This study has several limitations. First, unavoidable selection bias existed, and only CCLNs that underwent surgery were included. Second, the lymph nodes included in this study need to be first observed by the US, which depends on the location of lymph nodes and the diagnostic variation of sonographers, limiting their practicality. Third, the suspicious CCLNs observed by the sonographers did not receive individual attention from a pathologist. Thus, a large-scale study with a prospective design is needed to clarify these findings in the future.

## Conclusions

In this study, we described the various clinical and sonographic characteristics of metastatic CCLNs from patients with PTC, especially with respect to their differentiation from benign CCLNs in patients with PTC. Regarding the clinical characteristics of patients with PTC with CCLN metastasis, multifocality, and the absence of Hashimoto’s thyroiditis are independent clinical characteristics related to patients with PTC with malignant CCLNs. For preoperative US and CEUS, heterogeneous enhancement and centripetal perfusion are independent US features to identify malignant and benign CCLNs. Preoperative CEUS features can help distinguish malignant CCLNs from benign CCLNs to avoid unnecessary surgery or FNA.

## Data availability statement

The original contributions presented in the study are included in the article/supplementary material. Further inquiries can be directed to the corresponding author.

## Ethics statement

This study was reviewed and approved by the Ethics Committee of Second Xiangya Hospital, Central South University, China. The patients/participants provided their written informed consent to participate in this study.

## Author contributions

CN contributed to the conception and design of the work. FY participated to data analysis and manuscript writing. YG, KT, YX, RZ, SC, XL, QZ, LL, and ZZ participated to data collection and follow-up of patients. All authors contributed to the article and approved the submitted version.

## Funding

This project was funded by the National Natural Science Foundation of China (81974267), the Science and Technology Innovation Program of Hunan Province (2021RC3033), and the Hunan Provincial Natural Science Foundation of China (2022JJ30827).

## Conflict of interest

The authors declare that the research was conducted in the absence of any commercial or financial relationships that could be construed as a potential conflict of interest.

## Publisher’s note

All claims expressed in this article are solely those of the authors and do not necessarily represent those of their affiliated organizations, or those of the publisher, the editors and the reviewers. Any product that may be evaluated in this article, or claim that may be made by its manufacturer, is not guaranteed or endorsed by the publisher.
